# Supplementary material for: Research on an improved RT-DETR-based model for rice disease detection
Source: PLoS One. 2026 Jun 3;21(6):e0349237. doi: 10.1371/journal.pone.0349237 (PMC13232949; doi:10.1371/journal.pone.0349237)
Supplement: Attachment — Submitted filename: Response to Reviewers 2026.4.8.docx [file pone.0349237.s003.docx]

**Response to Reviewers**

Thank you for your valuable comments and suggestions on our manuscript. We have carefully considered each of your remarks and have made the necessary revisions to improve the quality and clarity of our work. Below, please find our detailed responses to each of your comments, along with the specific changes we have implemented in the manuscript.We appreciate your time and effort in reviewing our work.

**6. Review Comments to the Author**

**Please use the space provided to explain your answers to the questions above. You may also include additional comments for the author, including concerns about dual publication, research ethics, or publication ethics. (Please upload your review as an attachment if it exceeds 20,000 characters)**

**Reviewer #1:**

**1. In Table 5, the FPS values for the RTDETR-R101 and RTDETR-Co models are 3.6 and 9.5, respectively, which are relatively low compared to the baseline RTDETR-L's 10.6 FPS. It is suggested to briefly explain the reasons for this in the main text to help readers better understand these results.**

Response: Thank you for your valuable comments. We have added a brief explanation in Section 3.5.1 of the manuscript to clarify the reasons for the relatively low frame rates of the RTDETR-R101 and RTDETR-Co models. Specifically, the deeper architecture of ResNet-101 and the more complex feature extraction mechanisms in ConvNeXtV2 significantly increase the computational cost (e.g., higher floating-point operations and parameter counts), resulting in slower inference speeds compared to the baseline model RTDETR-L. This explanation has been incorporated into the revised manuscript to improve the clarity of the presentation.

**2. The discussion on the challenges of edge deployment in Section 5.3 is well addressed. If feasible optimization directions or technical approaches for the future could be briefly mentioned at the end of this section, it would provide readers with more forward-looking guidance and further strengthen the practical value of the paper.**

Response: Thank you for your valuable suggestions. We have added a brief discussion at the end of Section 5.3, outlining potential future research directions. These directions include model compression technologies (e.g., pruning, quantization, and knowledge distillation), hardware-aware optimization, neural architecture search, and deployment acceleration strategies for edge devices. This supplement enhances the practicality and forward-looking nature of this paper.

**Reviewer #2: The authors have made reasonable revisions in response to the review comments. The paper can be considered for acceptance.**

Response: Thank you！

**Reviewer #3: This study proposes ECL-RTDETR, an enhanced RT-DETR–based rice disease detection model.The authors have addressed my concerns. My decision is Accept.**

Response: Thank you！
